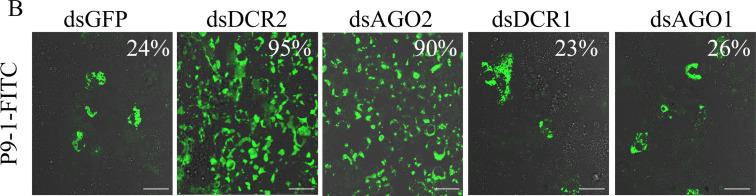# Correction for Lan et al., “Small Interfering RNA Pathway Modulates Initial Viral Infection in Midgut Epithelium of Insect after Ingestion of Virus”

**DOI:** 10.1128/jvi.00215-26

**Published:** 2026-03-05

**Authors:** Hanhong Lan, Hongyan Chen, Yuyan Liu, Chaoyang Jiang, Qianzhuo Mao, Dongsheng Jia, Qian Chen, Taiyun Wei

## AUTHOR CORRECTION

Volume 90, no. 2, p. 917–929, 2015, https://doi.org/10.1128/JVI.01835-15. Figure 2B should appear as shown in this correction. We regret that an incorrect panel for the dsAGO2 treatment was inadvertently included in the original. The figure has now been corrected, and this does not affect the conclusions of the study. We apologize for this error, which did not alter the final results.

**Fig 2:**